# Apolipoprotein B/A1 Ratio Is Associated with Severity of Coronary Artery Stenosis in CAD Patients but Not in Non-CAD Patients Undergoing Percutaneous Coronary Intervention

**DOI:** 10.1155/2021/8959019

**Published:** 2021-12-18

**Authors:** Rui Hua, Yijun Li, Wenyuan Li, Zhen Wei, Zuyi Yuan, Juan Zhou

**Affiliations:** ^1^Department of Cardiovascular Medicine, The First Affiliated Hospital, Xi'an Jiaotong University, Xi'an, Shaanxi, China; ^2^Department of Breast Surgery, The First Affiliated Hospital, Xi'an Jiaotong University, Xi'an, Shaanxi, China; ^3^Key Laboratory of Molecular Cardiology, Xi'an, Shaanxi, China; ^4^Key Laboratory of Environment and Genes Related to Diseases, Ministry of Education, Xi'an, Shaanxi, China

## Abstract

**Background and Aims:**

Lipid metabolism plays important roles in atherosclerosis. Several studies have found that lipoprotein is associated with coronary artery disease (CAD) and hyperlipidemia. Although the roles of the apolipoprotein B/A1 ratio (ApoB/A1) were originally thought to be atherosclerotic, few studies have focused on the specific relationship between ApoB/A1 and severity of coronary artery stenosis with or without the presence of CAD.

**Methods:**

A total of 6956 consecutive patients aged 21–98 years with suspected CAD who had undergone coronary angiography were enrolled. The severity of coronary lesions was evaluated using the Gensini score (GS). The relationships between ApoB/A1 and severity of coronary artery stenosis were evaluated.

**Results:**

A total of 1795 non-CAD patients and 5161 CAD patients were included in the observational analysis. Patients with CAD had higher ApoB/A1 than individuals without CAD (0.67 (0.53-0.82) vs. 0.61 (0.49-0.75), *p* < 0.001). In CAD patients, the higher the ApoB/A1 was, the higher the proportion of patients with MI, triple-vessel lesions, and higher Gensini scores. ApoB/A1 was significantly positively correlated with HbA1c and Gensini scores in CAD patients but not in non-CAD patients (all *p* < 0.001). Logistic analyses showed that ApoB/A1 could be a risk factor for multivessel disease (OR: 2.768, 95% CI: 1.868-4.103, *p* < 0.001). ApoB/A1 was found to be significantly positively correlated with the Gensini score in CAD patients.

**Conclusions:**

ApoB/A1 is highly associated with the presence and severity of coronary artery stenosis in patients with CAD but not in non-CAD patients.

## 1. Introduction

Coronary artery disease (CAD) is one of the leading causes of death and illness in developed countries and will soon become prominent throughout the world [1, 2]. As atherosclerosis is regarded as a lipid-driven inflammatory disease [3], dyslipidemia, including high low-density lipoprotein cholesterol (LDL-C) and triglyceride (TG) concentrations and low high-density lipoprotein cholesterol (HDL-C) concentrations, is a risk factor for CAD [4]. Although CAD patients with dyslipidemia are known to present with higher plaque burden as well as poorer prognosis [5–7], the underlying mechanism remains elusive and needs more effective serum prediction markers.

Apolipoproteins (Apo) are the primary structural proteins for lipoprotein particles that guide lipid transportation and facilitate lipid uptake and deposition into tissue, which play a central role in cholesterol metabolism [8]. Apolipoprotein genes have mutations, forming different allelic polymorphisms and further forming different phenotypes of apolipoproteins, which are mainly divided into five categories (A, B, C, D, and E) and affect the metabolism and utilization of lipids. Apolipoprotein B (ApoB) is the main component of very low-density lipoproteins (VLDL), intermediate-density lipoproteins (IDL), low-density lipoproteins (LDL), and lipoprotein(a), while ApoA1 represents the major high-density lipoprotein (HDL) Apo [9, 10].

ApoB/A1 can partially reflect the balance between atherogenic and atheroprotective cholesterol transport, independently associated with CAD [11]. It has also been reported that ApoB/A1 is associated with the risk of STEMI [12]. Moreover, ApoB/ApoA1 was proven to be a better risk predictor of atherosclerotic disease than the LDL/HDL [13, 14]. However, it remains unclear whether ApoB/A1 correlated with the severity of coronary artery stenosis, and the difference between CAD and non-CAD groups was still vague. According to the specific patient groups who would be diagnosed or treated by coronary angiography, we elucidated the relationship between ApoB/A1 and the severity of coronary artery stenosis.

## 2. Materials and Methods

### 2.1. Study Population

**Figure 1:**
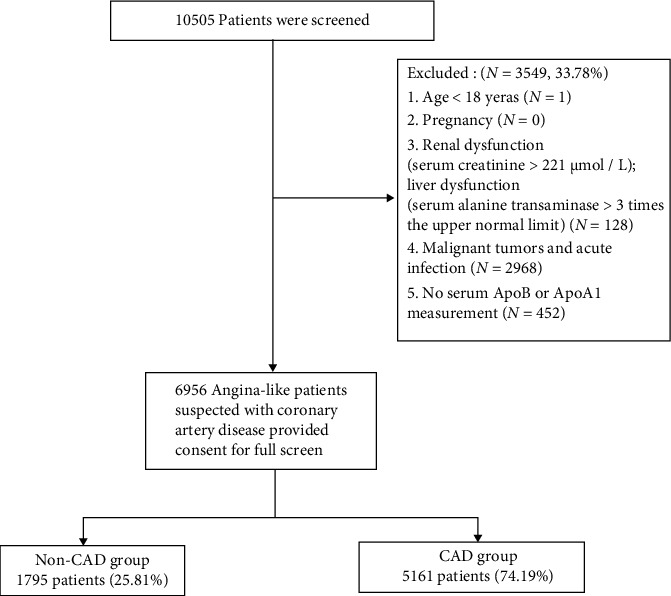
Study flowchart.

The study is a single-center, cross-sectional analysis. From April 2017 to March 2019, a total of 10505 consecutive patients with angina-like symptoms were admitted to the cardiology department of the First Affiliated Hospital of Xi'an Jiaotong University and then underwent coronary angiography (CAG) during hospitalization for suspicion of CAD. The inclusion criteria were as follows: (1) patients with angina-like symptoms were suspected with coronary artery disease and have undergone coronary angiography from April 2017 to March 2019 and (2) patients and family members were fully informed of their research content and signed an informed consent form. The exclusion criteria were (1) age < 18 years, (2) pregnancy, (3) renal dysfunction (serum creatinine > 221 *μ*mol/L) or liver dysfunction (serum alanine transaminase > 3 times the upper normal limit), (4) malignant tumors and acute infection, (5) no serum ApoB or ApoA1 measurement. A total of 6956 patients were ultimately enrolled in this study, and 5161 patients were diagnosed with CAD according to the CAG results as shown in Figure 1. The protocol was approved by the Ethics Committee of the First Affiliated Hospital of Xi'an Jiaotong University. Informed consent was obtained from all study participants.

### 2.2. Data Availability

Detailed demographic information, medical history, biochemical examination, drug usage, and angiographic data were obtained from the medical records. Hypertension was defined by two separate measures of blood pressure both higher than 140/90 mmHg or by a previous diagnosis when the patient was taking antihypertensive therapy [15]. Diabetes was confirmed by fasting glucose higher than 126 mg/dL or by a previous diagnosis when the patient was taking an oral hypoglycemic agent or insulin [16].

Whole-blood red blood cell distribution width (RDW) and hematocrit (Hct) data, plasma hemoglobin A1c (HbA1c) data, and serum total cholesterol (TC), triglyceride (TG), high-density lipoprotein cholesterol (HDL), low-density lipoprotein cholesterol (LDL), apolipoprotein B (ApoB), apolipoprotein A1 (ApoA1), alanine aminotransferase (ALT), gamma-glutamyl transpeptidase (GGT), total bile acids (TBA), homocysteine (Hcy), TnT-hs (hypersensitive troponin T), creatine kinase MB (CK-MB), N-terminal probrain natriuretic peptide (NT-pro-BNP), creatinine (Cr), cystatin C (Cys C), high-sensitivity C-reactive protein (Hs-CRP), fibrinogen (FIB), international normalized ratio (INR), D dimer (D-D), free triiodothyronine (FT3), triiodothyronine (T3), free thyroxine (FT4), and thyroxine (T4) were collected and measured using standard methods upon admission. Complete blood count was conducted by using blood analyzer BC5390 (Mindray, Shenzhen, China); a coagulation function test was conducted by using automatic hemagglutination apparatus CA7000 (Sysmex, Kobe, Japan); liver function and renal function tests were conducted by using automatic biochemical immune analyzer VITROS5600 (JNJ, New Jersey, US); blood lipid test was conducted by using a Hitachi biochemical analyzer TCSLST008AS-2 (Hitachi, Japan); thyroid function test was conducted by using cobas 8000 (Roche, Basel, Switzerland).

### 2.3. Coronary Angiograms and Scoring

Coronary angiography (CAG) was performed according to the standard Judkins techniques. The percentages of lumen stenosis were assessed by visual analyses. The diagnose of CAD is defined on the presence of at least 50% luminal diameter narrowing in at least one major coronary artery by two experienced interventional cardiologists [17]. The criteria of acute myocardial infarction (AMI) should meet typical chest pain for more than 30 minutes, elevated serum biomarkers, or typical features shown on the electrocardiogram. The number of narrow vessels is counted as the number of coronary arteries with ≥50% stenosis in three main branches, and left main coronary (LM) stenosis ≥ 50% is recorded as two diseased vessels. The severity of coronary lesions was assessed by the Gensini score according to a previously published method [18].

### 2.4. Statistical Method

All statistics were performed with SPSS software 25.0 (SPSS Inc., Chicago, IL, USA). Continuous variables were shown as the means ± standard deviations for normally distributed data or medians (25th, 75th percentiles) for nonnormally distributed data. The Kolmogorov–Smirnov test was used to assess the normal distribution of quantitative variables. Statistical comparisons were compared using *t*-tests or nonparametric tests among two groups as appropriate, and ANOVA and nonparametric tests were used among three groups. Categorical variables were expressed as frequencies and percentages, and the chi-square test was applied. The correlation between ApoB/A1 and CVD risk factors was analyzed using Spearman's correlation in non-CAD and CAD groups. The independent risk factors for multiple vessels were analyzed by logistic regression analyses. Adjusted *R*^2^ and 95% confidence intervals (CIs) were calculated by univariate and multivariate linear regression analysis models. All probability values were 2-tailed, and values of *p* < 5% were considered statistically significant.

## 3. Results

### 3.1. Baseline Data

From April 2017 to March 2019, 10505 patients with angina-like symptoms were enrolled in the study. After screening for inclusion and exclusion criteria, 1795 non-CAD patients and 5161 CAD patients were included in the analysis. Baseline patient characteristics were shown in Table 1 for patients with and without CAD. The median age was 62 (55-69) years in CAD patients and 60 (53-67) years in non-CAD patients. The median ApoB/A1 was 0.67 (0.53-0.82) in CAD patients and 0.61 (0.49-0.75) in non-CAD patients.

### 3.2. ApoB/A1 Was Associated with MI and the Severity of Coronary Lesions in CAD Patients

Patients diagnosed with CAD were divided into 3 groups based on ApoB/A1 tertiles, and a comparison of the clinical data of these groups was shown in Table 2. The proportions of male, baseline age, heart rate, TC, TG, LDL, HDL, ALT, GGT, Hcy, HbA1c, Hs-CRP, FIB, D-D, and N-terminal probrain natriuretic peptide levels all increased as the ApoB/A1 increased (all *p* < 0.05).

Moreover, the higher the ApoB/A1 was, the higher the proportion of patients with MI, triple-vessel lesions, and higher Gensini scores and the lower the proportion of patients with single-vessel lesions. The incidence of MI in ApoB/A1-H (34.9%) and ApoB/A1-M (23.0%) groups was higher than that in ApoB/A1-L (12.9%) group (*p* < 0.001). No significant difference in the proportion of diabetic patients or Cys C was observed among the three groups.

### 3.3. ApoB/A1 Was Associated with the Severity of Coronary Lesions in CAD Patients

Spearman's correlation analyses between ApoB/A1 and cardiovascular disease risk factors were shown in Table 3. The ApoB/A1 was inversely correlated with age, female sex, and HDL and positively correlated with TC, TG, LDL, and Hs-CRP in both non-CAD and CAD groups. Intriguingly, ApoB/A1 was only associated with HbA1c (*r* = 0.091, *p* < 0.001) and Gensini score (*r* = 0.188, *p* < 0.001) in CAD group, but not in non-CAD group.

### 3.4. The Predictive Value of ApoB/A1 for the Severity of Coronary Artery Stenosis in CAD Patients

We further explored whether ApoB/A1 contributed to the severity of coronary artery stenosis in CAD group.  Table 4 presented univariable logistic regression results for the number of vascular lesions associated with ApoB/A1. The increase in ApoB/ApoA1 was a risk factor for double-vessel (OR: 1.681, 95% CI: 1.230-2.296, *p* = 0.001) and triple-vessel (OR: 3.908, 95% CI: 2.900-5.268, *p* < 0.001) disease compared to single-vessel disease.

**Figure 2:**
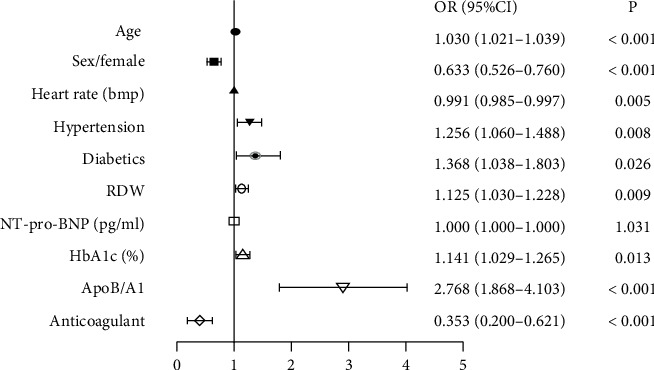
Risk factors for multivessel disease in CAD patients. RDW = red blood cell distribution width; NT-pro-BNP = N-terminal probrain natriuretic peptide; HbA1c = hemoglobin A1c; ApoB/A1 = apolipoprotein B/A1 ratio; OR = odds ratios; CI = confidence interval. ^∗^*p* < 0.05.

Multiple regression analysis was then utilized to further explain the association of ApoB/A1 and multiple diseased vessels. As shown in Table 5 and Figure 2, ApoB/A1 was found to be a significant risk factor for multidiseased vessels in CAD patients (OR: 2.768, 95% CI: 1.868-4.103, *p* < 0.001), after adjusting for age, female, RDW, Hct, TC, TG, HDL, LDL, ALT, GGT, TBA, Hcy, TnT-hs, CK-MB, NT-pro-BNP, Scr, Cys C, HbA1c, Hs-CRP, FIB, INR, D-D, FT3, T3, FT4, T4, and p2y12.

### 3.5. Association between ApoB/A1 and the Severity of Coronary Artery Stenosis

**Figure 3:**
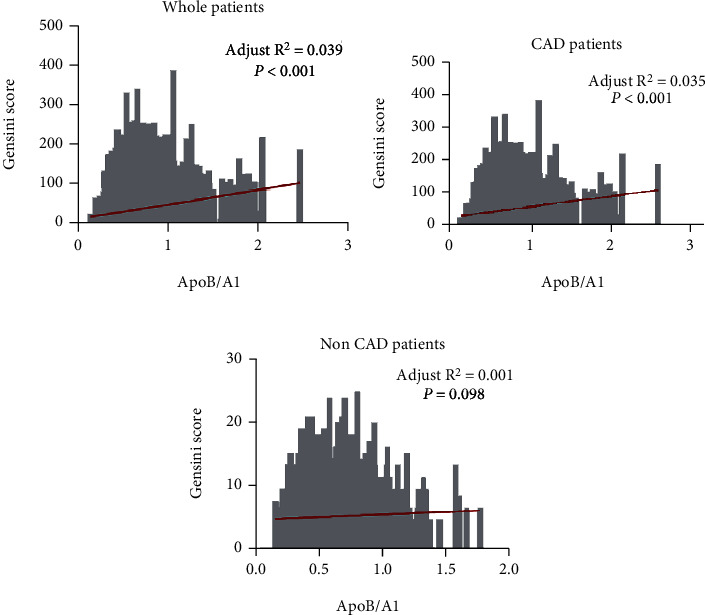
Simple linear analysis between ApoB/A1 and Gensini score in all patients and non-CAD and CAD patients. (a) Simple linear regression model with ApoB/A1 in relation to the Gensini score in the whole patient population. (b) Simple linear regression model with ApoB/A1 in relation to the Gensini score in non-CAD patients. (c) Simple linear regression model with ApoB/A1 in relation to the Gensini score in CAD patients. ApoB/A1 = apolipoprotein B/A1 ratio; CAD = coronary artery disease.

To investigate the relationship between ApoB/A1 and the severity of coronary artery stenosis, we utilized simple linear regression analysis (Table 6). The admission ApoB/A1 was found to be significantly positively correlated with the Gensini score by accumulating the area and severity of lesions in both the whole population (*R* square = 0.039; *β* (95%CI) = 0.198 (0.175 to 0.221), *p* < 0.001) and CAD patients (*R* square = 0.034; *β* (95%CI) = 0.185 (0.158 to 0.212, *p* < 0.001) (Figure 3, Table 6).

Multiple regression analysis was then utilized to further determine the association of the severity of coronary artery lesions and baseline characteristics, including ApoB/A1. Notably, only the ApoB/A1 was found to be significantly positively correlated with the severity of coronary artery stenosis in CAD patients (95% CI 0.103 to 0.181, *p* < 0.001), not in the group of non-CAD patients (95% CI −0.007 to 0.136, *p* = 0.079) (Table 7).

## 4. Discussion

In the current study, we noted that ApoB/A1 was higher in CAD patients than in non-CAD patients. The higher the ApoB/A1 was, the higher the proportion of patients with MI, triple-vessel lesions, and higher Gensini scores. In CAD patient population, ApoB/A1 was related to the Gensini score and Hs-CRP, and logistic regression analysis demonstrated that elevated ApoB/A1 was an important and independent predictor of multivessel disease. Moreover, linear regression analysis indicated that the ApoB/A1 was significantly positively correlated with the Gensini score, an indicator of the severity of coronary artery stenosis.

The important implication of the present study is that ApoB/A1 is identified as a serum predictor for the severity of cardiovascular lesions in CAD patients. Notably, our study is the first to show a positive linear correlation between ApoB/A1 and Gensini score in patients with CAD but not in non-CAD patients, indicating that ApoB/A1 has a potential mechanism for the progression of coronary lesions in CAD patients. Moreover, a significant correlation between ApoB/A1 and other cardiovascular risk factors is identified, implying that ApoB/A1 monitoring might be related to inflammatory pathology. A previous study indicated that the ApoB/A1 could predict the vulnerability of LMCA plaques [19]. It is also known that ApoB/A1 is predictive of the severity of CAD, providing more prognostic information than other routine lipid profiles [20]. However, few studies have addressed the question of whether ApoB/A1 is related to the severity of coronary disease using a complex and systemic score, i.e., the Gensini score or SYNTAX score to predict the severity of coronary artery stenosis more accurately. Although coronary angiography is the gold standard to diagnose CAD and its severity, detecting ApoB/A1 also has several advantages in CAD patients; for example, it is noninvasive, simpler, and cheaper compared with coronary angiography. It is still applicable to some patients with contraindications to coronary angiography.

In our study, we have illustrated the relationship of ApoB/A1 and severity of coronary artery stenosis in detail as compared to other work previously. Lipids are transported by lipoproteins, which consist of lipids, including cholesterol, triglycerides, phospholipids, and apolipoproteins. ApoA1 and ApoB are two major types of Apos, reflecting the balance between the opposing processes of cholesterol transport [21]. In recent years, high levels of ApoB and low levels of ApoA1 have been found to be associated with the higher risk of cardiovascular disease, metabolic syndrome, ischemic stroke, and Alzheimer's disease [14, 21–23].

There is conflicting evidence regarding ApoB/A1 with oxidative stress and endothelial dysfunction in statin-treated patients with coronary artery disease [24]. It has been widely recognized that a higher level of LDL contributes to the development of CAD in a previous cohort [25]. However, other lipid markers, such as very low-density lipoprotein, intermediate-density lipoprotein, and Lp(a), are needed to evaluate residual atherosclerotic risk, and ApoB might be a better lipid marker than LDL, as it consists of these atherosclerotic lipoproteins. ApoA1 also reverses cholesterol as an antioxidant and anti-inflammatory agent.

This study have several limitations that should be mentioned. First, observational cross-sectional studies are less convincing than clinical trial studies, and a causal relationship cannot be established. Second, this is a single-center study, and different regions and ethnic groups are needed to support our findings in further studies; the correlation of ApoB/A1 and the progression of CAD in the mechanism is unknown, and further mechanistic research is needed prospectively. Third, as these patients were suspected with CAD and the prescription rates of aspirin, clopidogrel, and statin were higher than those of normal patients, this may affect their laboratory examination. Last but not least, there is a statistically significant difference in age and gender between CAD and non-CAD group, which can be a handicap that can affect the final results. According to our analysis, the non-CAD group has 49.4% hypertension, which can also be a limitation of our analysis.

In conclusion, from these sizable Chinese cohort results, our study is the first to indicate that patients with CAD had higher ApoB/A1 than individuals in non-CAD group. Moreover, ApoB/A1 is highly and independently associated with the severity of coronary artery stenosis of CAD patients but not in non-CAD patients. Therefore, ApoB/A1, considered a marker of lipid metabolism, may serve as a clinically useful and low-cost biomarker to help predict CAD and its severity and may provide guidance for the clinical treatment of CAD.

## 5. Conclusions

ApoB/A1 is highly associated with the presence and severity of coronary artery stenosis in patients with CAD but not in non-CAD patients.

## Figures and Tables

**Table 1:** Basic characteristics of the patients with CAD and without CAD.

Variables	Whole (*n* = 6956)	Non-CAD (*n* = 1795)	CAD (*n* = 5161)	*p*
ApoB/A1	0.65 (0.52-0.80)	0.61 (0.49-0.75)	0.67 (0.53-0.82)	<0.001^∗^
Sex (female)	2503 (36)	897 (50.0)	1606 (31.1)	<0.001^∗^
Age (year)	62 (54-68)	60 (53-67)	62 (55-69)	<0.001^∗^
Heart rate (bmp)	74 (66-82)	74 (66-82)	74 (66-82)	0.466
Hypertension	3881 (55.8)	886 (49.4)	2995 (58.0)	<0.001^∗^
Diabetics	1523 (21.9)	243 (13.5)	1280 (24.8)	<0.001^∗^
Smoking history	2878 (41.4)	534 (29.7)	2344 (45.4)	<0.001^∗^
RDW (%)	12.8 (12.4-13.3)	12.8 (12.4-13.3)	12.9 (12.4-13.3)	0.514
Hct (%)	41.8 (38.9-44.7)	41.5 (38.6-44.5)	41.9 (38.9-44.7)	0.057
TC (mmol/L)	3.77 (3.16-4.46)	3.79 (3.19-4.44)	3.77 (3.15-4.47)	0.528
TG (mmol/L)	1.30 (0.95-1.83)	1.26 (0.93-1.74)	1.32 (0.96-1.85)	0.001^∗^
HDL (mmol/L)	0.96 (0.82-1.13)	1.00 (0.85-1.19)	0.94 (0.81-1.11)	<0.001^∗^
LDL (mmol/L)	2.12 (1.63-2.7)	2.13 (1.61-2.69)	2.12 (1.63-2.70)	0.860
ApoB (g/L)	0.72 (0.59-0.88)	0.71 (0.57-0.85)	0.73 (0.59-0.88)	<0.001^∗^
ApoA1 (g/L)	1.11 (0.99-1.25)	1.16 (1.03-1.29)	1.10 (0.98-1.23)	<0.001^∗^
ALT (U/L)	22 (15-33)	19 (14-29)	23 (16-35)	<0.001^∗^
GGT (U/L)	22 (15-35)	20 (14-33)	23 (16-35)	<0.001^∗^
TBA (*μ*mol/L)	3.7 (2.2-6.0)	4.0 (2.4-6.6)	3.6 (2.2-5.9)	<0.001^∗^
Hcy (*μ*mol/L)	16.30 (12.80-22.00)	15.20 (12.20-19.78)	16.70 (13.10-22.80)	<0.001^∗^
TnT-hs (ng/mL)	0.01 (0-0.03)	0.01 (0-0.01)	0.01 (0.01-0.06)	<0.001^∗^
CK-MB (U/L)	12 (8-17)	11 (8-15)	12 (9-18)	<0.001^∗^
NT-pro-BNP (pg/mL)	122.95 (50.48-378.10)	81.55 (37.05-197.30)	144.50 (58.12-481.45)	<0.001^∗^
Scr (*μ*mol/L)	62 (53-73)	60 (50-71)	63 (53-74)	<0.001^∗^
Cys C (mg/L)	0.83 (0.64-1.08)	0.82 (0.62-1.06)	0.84 (0.65-1.09)	<0.001^∗^
HbA1c (%)	5.70 (5.40-6.20)	5.60 (5.30-5.90)	5.70 (5.40-6.30)	<0.001^∗^
Hs-CRP (mmol/L)	1.20 (0.55-3.59)	0.87 (0.40-2.06)	1.42 (0.62-4.31)	<0.001^∗^
FIB (g/L)	3.00 (2.56-3.53)	2.85 (2.45-3.32)	3.05 (2.61-3.63)	<0.001^∗^
INR	1.02 (0.97-1.06)	1.01 (0.96-1.05)	1.02 (0.97-1.07)	<0.001^∗^
D-D (mg/L)	0.45 (0.30-0.65)	0.40 (0.30-0.60)	0.49 (0.30-0.70)	<0.001^∗^
FT3 (pmol/L)	4.37 (3.79-5.00)	4.44 (3.83-5.12)	4.35 (3.77-4.95)	<0.001^∗^
T3 (pmol/L)	1.10 (0.94-1.28)	1.11 (0.96-1.29)	1.09 (0.93-1.27)	0.002^∗^
FT4 (pmol/L)	14.6 (12.5-16.9)	14.9 (12.6-17.2)	14.6 (12.4-16.8)	0.004^∗^
T4 (pmol/L)	7.47 (6.22-8.89)	7.53 (6.28-8.96)	7.45 (6.2-8.88)	0.218
Aspirin	6765 (97.3)	1725 (96.1)	5040 (97.7)	0.001^∗^
P2y12	6740 (96.9)	1713 (95.4)	5027 (97.4)	<0.001^∗^
Statin	6729 (96.7)	1696 (94.5)	5033 (97.5)	<0.001^∗^
Anticoagulant	163 (2.3)	52 (2.9)	111 (2.2)	0.085
Gensini score	25.00 (8.00-58.00)	5.00 (2.00-8.00)	40.00 (20.00-72.00)	<0.001^∗^

Data are presented as the medians (25th, 75th percentiles) or number (%). ApoB/A1, age, heart rate, RDW, Hct, TC, TG, HDL, LDL, ApoB, ApoA1, ALT, GGT, TBA, Hcy, TnT-hs, CK-MB, NT-pro-BNP, Scr, Cys C, HbA1c, Hs-CRP, FIB, INR, D-D, FT3, T3, FT4, T4, and Gensini score were given as medians (25th, 75th percentiles); sex, hypertension, diabetics, smoking history, and usage of aspirin, p2y12, statin, and anticoagulant were given as number (%) (^∗^*p* < 0.05). RDW = red blood cell distribution width; Hct = hematocrit; TC = total cholesterol; TG = triglyceride; HDL = high-density lipoprotein cholesterol; LDL = low-density lipoprotein cholesterol; ApoB = apolipoprotein B; ApoA1 = apolipoprotein A1; ALT = alanine aminotransferase; GGT = gamma-glutamyl transpeptidase; TBA = total bile acids; Hcy = homocysteine; TnT-hs = hypersensitive troponin T; CK-MB = creatine kinase MB; NT-pro-BNP = N-terminal probrain natriuretic peptide; Scr = serum creatinine; Cys C = cystatin C; HbA1c = hemoglobin A1c; Hs-CRP = high-sensitivity C-reactive protein; FIB = fibrinogen; INR = international normalized ratio; D-D = D-dimer; FT3 = free triiodothyronine; T3 = triiodothyronine; FT4 = free thyroxine; T4 = thyroxine; p2y12 = platelet p2y12 antagonist; CAD = coronary artery disease.

**Table 2:** Comparison of clinical data between groups in CAD patients with different level of ApoB/A1.

Variables	ApoB/A1-L (0.14-0.57) *N* = 1719	ApoB/A1-M (0.57-0.76) *N* = 1718	ApoB/A1-H (0.76-2.45) *N* = 1724	*p*
Sex/female	631 (36.7)	542 (31.5)	433 (25.1)	<0.001^∗^
Age (year)	64 (57-71)	62 (55-69)	60 (52-67)	<0.001^∗^
Heart rate (bmp)	72 (64-80)	73 (66-81)	75 (67-84)	<0.001^∗^
Hypertension	1033 (60.1)	1023 (59.5)	939 (54.5)	0.001^∗^
Diabetics	408 (23.7)	414 (24.1)	458 (26.6)	0.112
Smoking history	696 (40.5)	765 (44.5)	883 (51.2)	<0.001^∗^
TC (mmol/L)	3.14 (2.73-3.65)	3.77 (3.28-4.30)	4.46 (3.90-5.14)	<0.001^∗^
TG (mmol/L)	1.03 (0.79-1.39)	1.33 (1.01-1.87)	1.64 (1.23-2.26)	<0.001^∗^
HDL (mmol/L)	1.04 (0.88-1.21)	0.94 (0.80-1.09)	0.88 (0.77-1.02)	<0.001^∗^
LDL (mmol/L)	1.54 (1.25-1.89)	2.15 (1.81-2.55)	2.81 (2.36-3.36)	<0.001^∗^
ApoB (g/L)	0.55 (0.47-0.63)	0.73 (0.66-0.82)	0.92 (0.83-1.04)	<0.001^∗^
ApoA1 (g/L)	1.19 (1.06-1.32)	1.10 (0.99-1.23)	1.01 (0.91-1.13)	<0.001^∗^
ALT (U/L)	22 (15-32)	23 (16-35)	24 (16-37)	<0.001^∗^
GGT (U/L)	20 (14-31)	23 (16-34)	26 (18-42)	<0.001^∗^
TBA (*μ*mol/L)	3.80 (2.30-5.80)	3.60 (2.28-6.10)	3.30 (2.00-5.60)	<0.001^∗^
Hcy (*μ*mol/L)	16.00 (12.40-21.78)	16.55 (13.30-22.48)	17.50 (13.50-24.20)	<0.001^∗^
TnT-hs (ng/mL)	0.01 (0.01-0.02)	0.01 (0.01-0.06)	0.02 (0.01-0.20)	<0.001^∗^
CK-MB (U/L)	12 (8-17)	11 (8-15)	12 (9-18)	<0.001^∗^
NT-pro-BNP (pg/mL)	134.40 (63.12-371.30)	140.30 (56.42-484.70)	163.30 (55.18-549.40)	0.011^∗^
Scr (*μ*mol/L)	62 (52-73)	63 (53-73)	64 (55-76)	<0.001^∗^
Cys C (mg/L)	0.84 (0.64-1.09)	0.84 (0.65-1.08)	0.85 (0.66-1.09)	0.820
HbA1c (%)	5.7 (5.4-6.2)	5.7 (5.4-6.2)	5.8 (5.5-6.5)	<0.001^∗^
Hs-CRP (mg/L)	0.91 (0.42-2.39)	1.35 (0.65-3.62)	2.39 (0.94-8.16)	<0.001^∗^
FIB (g/L)	2.92 (2.50-3.40)	3.04 (2.62-3.56)	3.23 (2.73-3.97)	<0.001^∗^
D-D (mg/L)	0.44 (0.30-0.60)	0.45 (0.30-0.62)	0.50 (0.31-0.70)	<0.001^∗^
AMI	221 (12.9)	395 (23.0)	602 (34.9)	<0.001^∗^
Single-vessel	711 (41.4)	596 (34.7)	545 (31.6)	<0.001^∗^
Double-vessel	513 (29.8)	562 (32.7)	506 (29.4)	
Triple-vessel	495 (28.8)	560 (32.6)	673 (39.0)	
Gensini	32.00 (17.00-60.00)	42.00 (21.00-71.25)	49.00 (25.00-82.00)	<0.001^∗^

Patients were divided into three ApoB/A1-L, ApoB/A1-M, and ApoB/A1-H groups based on the ApoB/A1 level. Data are presented as the medians (25th, 75th percentiles) or number (%). Age, heart rate, RDW, Hct, TC, TG, HDL, LDL, ApoB, ApoA1, ALT, GGT, TBA, Hcy, TnT-hs, CK-MB, NT-pro-BNP, Scr, Cys C, HbA1c, Hs-CRP, FIB, D-D, and Gensini score were given as medians (25th, 75th percentiles); sex, hypertension, diabetics, smoking history, AMI, single-vessel, double-vessel, and triple-vessel were given as number (%) (^∗^*p* < 0.05). TC = total cholesterol; TG = triglyceride; HDL = high-density lipoprotein cholesterol; LDL = low-density lipoprotein cholesterol; ApoB = apolipoprotein B; ApoA1 = apolipoprotein A1; ALT = alanine aminotransferase; GGT = gamma-glutamyl transpeptidase; TBA = total bile acids; Hcy = homocysteine; TnT-hs = hypersensitive troponin T; CK-MB = creatine kinase MB; NT-pro-BNP = N-terminal probrain natriuretic peptide; Scr = serum creatinine; Cys C = cystatin C; HbA1c = hemoglobin A1c; Hs-CRP = high-sensitivity C-reactive protein; FIB = fibrinogen; D-D = D-dimer; AMI = acute myocardial infarction; CAD = coronary artery disease.

**Table 3:** The relationship between ApoB/A1 and cardiovascular risk factors by Spearman's analysis.

	Non-CAD		CAD	
	*r*	*p*	*r*	*p*
Age (year)	-0.252	<0.001^∗^	-0.172	<0.001^∗^
Sex/female	-0.137	<0.001^∗^	-1.118	<0.001^∗^
TC (mmol/L)	0.581	<0.001^∗^	0.608	<0.001^∗^
TG (mmol/L)	0.354	<0.001^∗^	0.411	<0.001^∗^
HDL (mmol/L)	-0.356	<0.001^∗^	-0.309	<0.001^∗^
LDL (mmol/L)	0.721	<0.001^∗^	0.731	<0.001^∗^
HbA1c (%)	0.034	0.158	0.091	<0.001^∗^
Hs-CRP (mmol/L)	0.166	<0.001^∗^	0.294	<0.001^∗^
Gensini score	0.025	0.283	0.188	<0.001^∗^

TC = total cholesterol; TG = triglyceride; HDL = high-density lipoprotein cholesterol; LDL = low-density lipoprotein cholesterol; HbA1c = hemoglobin A1c; Hs-CRP = high-sensitivity C-reactive protein; FIB = fibrinogen; INR = international normalized ratio; D-D = D-dimer; FT3 = free triiodothyronine; T3 = triiodothyronine; FT4 = free thyroxine; T4 = thyroxine; p2y12 = platelet p2y12 antagonist; CAD = coronary artery disease. ^∗^*p* < 0.05.

**Table 4:** Odds ratios of double- and triple-diseased vessels in relation to ApoB/A1 levels in CAD patients.

	OR	95% CI	SEM	*p*
Single-vessel	Reference			
Double-vessel	1.681	1.230-2.296	0.159	0.001
Triple-vessel	3.908	2.900-5.268	0.152	<0.001

OR: odds ratios; CI: confidence interval; SEM: standard error of measurement. ^∗^*p* < 0.05.

**Table 5:** Odds ratios of multiple diseased vessels in relation to ApoB/A1.

	OR	95% CI	SEM	*p*
ApoB/A1	2.768	1.868-4.103	0.201	<0.001
Age (year)	1.030	1.021-1.039	0.004	<0.001
Sex/female	0.633	0.526-0.760	0.094	<0.001
Heart rate (bmp)	0.991	0.985-0.997	0.003	0.005
Hypertension	1.256	1.060-1.488	0.086	0.008
Diabetes	1.368	1.038-1.803	0.141	0.026
RDW (%)	1.125	1.030-1.228	0.045	0.009
NT-pro-BNP (pg/mL)	1.000	1.000-1.000	0.000	1.031
HbA1c (%)	1.141	1.029-1.265	0.053	0.013
Anticoagulant	0.353	0.200-0.621	0.289	<0.001

RDW = red blood cell distribution width; NT-pro-BNP = N-terminal probrain natriuretic peptide; HbA1c = hemoglobin A1c; ApoB/A1 = apolipoprotein B/A1 ratio; OR = odds ratios; CI = confidence interval; SEM = standard error of measurement. ^∗^*p* < 0.05.

**Table 6:** Linear regression analysis of ApoB/A1 and coronary artery stenosis in all patients and non-CAD and CAD patients.

Variable	Adjust *R*^2^	Coefficient	95% CI	SEM	*p*
Whole	0.039	0.198	0.175-0.221	0.012	<0.001^∗^
Non-CAD	0.001	0.039	-0.007-0.085	0.024	0.098
CAD	0.034	0.185	0.158-0.212	2.486	<0.001^∗^

CI: confidence interval; SEM: standard error of measurement. ^∗^*p* < 0.05.

**Table 7:** Multiple regression analysis of coronary artery stenosis in patients (a) without CAD and (b) with CAD.

Variable	Coefficient	95% CI	SEM	*p*
(a) Non-CAD				
ApoB/A1	0.064	-0.007 to 0.136	0.037	0.079
Age (year)	0.144	0.077 to 0.212	0.034	<0.001^∗^
HDL (mmol/L)	-0.064	-0.134 to 0.006	0.036	0.073
HbA1c (%)	0.056	-0.008 to 0.119	0.032	0.086
Hs-CRP (mg/L)	0.008	-0.056 to 0.072	0.033	0.807
Hs-TNT (ng/ml)	0.008	-0.055 to 0.071	0.032	0.795
TBA (*μ*mol/L)	0.017	-0.046 to 0.080	0.032	0.589
Scr (*μ*mol/L)	0.046	-0.018 to 0.110	0.033	0.162
FT4 (pmol/L)	0.014	-0.050 to 0.078	0.032	0.664

(b) CAD				
ApoB/A1	0.142	0.103 to 0.181	0.020	<0.001^∗^
Age (year)	0.08	0.043 to 0.117	0.019	<0.001^∗^
HDL (mmol/L)	-0.043	-0.080 to 0.005	0.019	0.027^∗^
HbA1c (%)	0.122	0.087 to 0.158	0.018	<0.001^∗^
Hs-CRP (mg/L)	0.042	0.003 to 0.081	0.02	0.037^∗^
Hs-TNT (ng/mL)	0.058	0.020 to 0.096	0.019	0.003^∗^
TBA (*μ*mol/L)	-0.037	-0.072 to -0.001	0.018	0.041^∗^
Scr (*μ*mol/L)	0.075	0.043 to 0.117	0.019	<0.001^∗^
FT4 (pmol/L)	-0.041	-0.077 to -0.006	0.018	0.023^∗^

HDL = high-density lipoprotein cholesterol; TBA = total bile acids; TnT-hs = hypersensitive troponin T; Scr = serum creatinine; Cys C = cystatin C; HbA1c = hemoglobin A1c; Hs-CRP = high-sensitivity C-reactive protein; FT4 = free thyroxine; ApoB/A1 = apolipoprotein B/A1; CAD = coronary artery disease; CI = confidence interval; SEM = standard error of measurement. Adjusted for age, HDL, HbA1c, Hs-CRP, TnT-hs, TBA, Scr, FT4, and ApoB/A1. ^∗^*p* < 0.05.

## Data Availability

Data supporting the conclusions of this article are included within the article and available from the corresponding authors on reasonable request.
